# KCNE1 does not shift TMEM16A from a Ca^2+^ dependent to a voltage dependent Cl^-^ channel and is not expressed in renal proximal tubule

**DOI:** 10.1007/s00424-023-02829-5

**Published:** 2023-07-13

**Authors:** Khaoula Talbi, Jiraporn Ousingsawat, Raquel Centeio, Rainer Schreiber, Karl Kunzelmann

**Affiliations:** Physiological Institute, University of Regensburg, University street 31, D-93053 Regensburg, Germany

**Keywords:** TMEM16A, KCNE1, KCNQ1, Channel regulation, Voltage dependence, Ca^2+^ activated Cl^-^ channel

## Abstract

The TMEM16A (ANO1) Cl^-^ channel is activated by Ca^2+^ in a voltage-dependent manner. It is broadly expressed and was shown to be also present in renal proximal tubule (RPT). KCNQ1 is an entirely different K^+^ selective channel that forms the cardiac I_KS_ potassium channel together with its ß-subunit KCNE1. Surprisingly, KCNE1 has been claimed to interact with TMEM16A, and to be required for activation of TMEM16A in mouse RPT. Interaction with KCNE1 was reported to switch TMEM16A from a Ca2^2+^-dependent to a voltage-dependent ion channel. Here we demonstrate that KCNE1 is not expressed in mouse RPT. TMEM16A expressed in RPT is activated by angiotensin II and ATP in a KCNE1-independent manner. Coexpression of KCNE1 does not change TMEM16A to a voltage gated Cl^-^ channel and Ca^2+^-dependent regulation of TMEM16A is fully maintained in the presence of KCNE1. While overexpressed KCNE1 slightly affects Ca^2+^-dependent regulation of TMEM16A, the data provide no evidence for KCNE1 being an auxiliary functional subunit for TMEM16A.

**Supplementary Information:**

The online version contains supplementary material available at 10.1007/s00424-023-02829-5.

## Introduction

KCNQ1 (K_v_LQT1; Kv7.1) and IsK (MinK; KCNE1) associate to form the delayed time-dependent I_Ks_ cardiac potassium channel [3, 27]. The I_Ks_ channel complex is central for repolarization of the heart muscle action potential. Co-assembly of KCNQ1 with different accessory proteins of the KCNE family results in different phenotypic K^+^ currents. When co-assembled with KCNE1, this single membrane spanning peptide of 129 residues causes KCNQ1 to operate as a slowly activating delayed rectifier K^+^ current (I_Ks_). When co-assembled with KCNE2 or KCNE3, KCNQ1 channels are constitutively open [29] and are further activated by an increase in intracellular cAMP [17, 25]. While KCNQ1/KCNE2/3 currents show little time and voltage dependence, I_KS_ is strongly time dependent and outwardly rectifying.

The Ca^2+^ activated Cl^-^ channel TMEM16A has an entirely different structure compared to KCNQ1 and operates as a stable dimer [22]. At 20°C it is closed at basal intracellular Ca^2+^ concentrations, but partially open at higher temperatures [10, 30, 39]. Increase in intracellular Ca^2+^ opens TMEM16A in a voltage-dependent manner [24, 38]. Comprehensive studies identified two main Ca^2+^ binding sites in each subunit of TMEM16A along with additional sites critical for channel gating [20, 24, 36]. Cryo-EM structures of TMEM16A revealed the mechanisms for Ca^2+^ binding, transition from closed to open state and anion conduction [11, 22, 23]. Conformational changes induced by Ca^2+^ binding and subsequent rearrangements lead to opening of the pore [11, 19]. Additional control of gating is provided by phosphatidylinositol bisphosphate (PIP_2_), calmodulin and calmodulin-dependent kinase [21, 35, 40].

An earlier report showed that expression of KCNE1 in *Xenopus* oocytes leads to the appearance of endogenous Cl^-^ currents in *Xenopus* oocytes [1] and a subsequent recent study reported interaction of KCNE1 with TMEM16A [2]. It was claimed that KCNE1 switches TMEM16A to a voltage-dependent channel that does no longer require intracellular Ca^2+^ to be activated. Moreover, Ávalos Prado et al presented evidence for KCNE1-dependent angiotensin II-regulated TMEM16A in mouse proximal tubular epithelial cells [2].

In contrast to these findings, we demonstrate in the present study that i) KCNE1 does not switch overexpressed or endogenous TMEM16A from a Ca^2+^ dependent to a purely voltage-dependent ion channel. In the presence of KCNE1, TMEM16A still requires intracellular Ca^2+^ to be activated. ii) Neither time-dependent nor voltage-dependent activation of TMEM16A is affected by KCNE1. iii) An extracellular KCNE1 peptide only transiently activates overexpressed, but not endogenous TMEM16A, without affecting time-dependence. iv) KCNE1 is not expressed in mouse renal proximal tubule (RPT) and does not affect activation of TMEM16A in RPT cells. v) KCNE1 and KCNE1-peptide marginally affect Ca^2+^-dependent activation of TMEM16A, probably due to non-specific charge interaction.

## Methods

### Cell culture and primary cells

Human embryonic kidney 293T (HEK293T) cells were maintained at 37 °C/5% CO_2_ in DMEM media supplemented with 2 mM L-glutamine and 10% fetal bovine serum (FBS). HEK293T cells were transfected using a standard protocol for Lipofectamine3000 (Thermo Fisher Scientific). Experiments were carried out 24–48 h after transfection. Human cystic fibrosis bronchial epithelial (CFBE) cells were cultured at 37 °C/5% CO_2_ in MEM media supplemented with 10% FBS. HEK293 and CFBE cells were seeded on glass coverslips for patch clamp experiments. BCi-NS1 cells (kindly provided by Prof. R. Crystal, Weill Cornell Medical College, New York, USA) were cultured in supplemented Bronchial Epithelial Cell Growth Medium (BEGM; Lonza) at 37 °C/5% CO_2_. BCi-NS1 cells were polarized on permeable supports (Snapwell^TM^; Corning) for 30 days.

Primary proximal tubular epithelial cells were isolated from wild-type mice as described previously [8] and cultured at 37 °C/5% CO_2_ in DMEM/ F12 supplemented with 1% FBS, 1% Pen/Strep, 1% L-Glutamine (200 mM), 1% ITS (100×), 50 nM hydrocortisone, 5 nM triiodothyronine, and 5 nM Epidermal Growth Factor (Sigma). Cells were seeded on collagen coated glass coverslips and used for experiments 48-72h after transfection.

### Patch clamp

Cells were patch clamped when grown on coated glass coverslips and experiments were performed at 37°C. Patch pipettes were filled with a cytosolic-like solution containing (in mM): KCl 30, K-Gluconate 95, NaH_2_PO_4_ 1.2, Na_2_HPO_4_ 4.8, EGTA 1, Ca-Gluconate 0.758, MgCl_2_ 1.03, D-Glucose 5, ATP 3; pH 7.2. If not indicated otherwise, the intracellular (pipette) Ca^2+^ activity was 0.1 μM. The bath was perfused continuously with Ringer’s solution (in mM): NaCl 145, KH_2_PO_4_ 0.4, K_2_HPO_4_ 1.6, Glucose 5, MgCl_2_ 1, Ca-Gluconate 1.3) at a rate of 6 mL/min. Patch pipettes had an input resistance of 2–5 MΩ and whole cell currents were corrected for serial resistance. The current/voltage (I/V) relationship was determined by pulsing from the holding potential of -100 mV to test potentials between −100 and +100 mV increasing in 20 mV increments. Currents were recorded using the EPC-9 computer-controlled amplifier, and PULSE software (HEKA) as well as Chart software (AD Instruments).

### Transepithelial Ussing chamber recordings

Polarized BCi-NS1 cells were measured under short-circuit conditions in non-perfused, internal fluid circulation Ussing chambers (Physiologic Instruments). Cells were bathed symmetrically with 5 mL bicarbonate-buffered Ringer solution (mmol/l: NaCl 118.75; KH_2_PO_4_ 0,4; K_2_HPO_4_ 1,6; Glucose 5; MgSO_4_ 1; Ca-Gluconate 1.3, NaHCO_3_ 25; bubbled with 95% O_2_/5% CO_2_; pH 7.4) and kept at 37°C by a circulating water bath system. Stimulation with compounds was performed on either the apical or basolateral sides via direct stock solution dilution in the respective hemichamber fluid. The transepithelial voltage (V_te_) referring to the basolateral side was measured and short-circuited to 0 mV with a voltage clamp (VCC MC6-2S; Physiologic Instruments) connected to the chambers through Ag/AgCl electrodes and agar bridges (3-4% agar in 3M KCl). The offset between voltage electrodes and the system fluid resistance was compensated before cell mounting. The short-circuit current (*I*_*sc*_) was recorded using the Acquire&Analyze II data acquisition system (Physiologic Instruments). Transport inhibition or stimulation for each compound was calculated as the difference between the respective currents before and after compound addition (Δ*I*_*sc*_).

### Measurement of intracellular Ca^2+^

The plasma membrane bound calcium sensor GCaMP6 is a calcium indicator consisting of circularly permuted green fluorescent protein (cpGFP), the calcium-binding protein calmodulin (CaM), and CaM-interacting M13 peptide. Calcium binding induces conformational changes in the CaM/M13 complex causing increased brightness. HEK293T cells were transfected on coated glass coverslips with pGP-CMV-GCaMP6s. Fluorescence ratio at 485/405 nm was measured after 48h in cells perfused with Ringer’s solution at 37°C using an inverted microscope (Axiovert S100; Carl Zeiss Microscopy) and a high speed polychromator system (VisiChrome). Measurements of global cytosolic Ca^2+^ concentration were performed on cells loaded with 2 μM Fura-2/AM and 0.02% Pluronic F-127 (Thermo Fisher) in ringer solution for 1 h at room temperature. The results were obtained at 340/380 nm fluorescence ratio (after background subtraction). The formula used to calculate [Ca^2+^]_i_ was

$${\left[{\textrm{Ca}}^{2+}\right]}_{\textrm{i}}= Kd\times \left(R- Rmin\right)/\left( Rmax-R\right)\times \left( Sf2/ Sb2\right)$$

where R is the observed fluorescence ratio. The values Rmax and Rmin (maximum and minimum ratios) and the constant Sf2/Sb2 (fluorescence of free and Ca^2+^-bound Fura-2 at 380 nm) were calculated using 1 μM ionomycin (Cayman), 5 μM nigericin, 10 μM monensin (Sigma-Aldrich). The dissociation constant for the Fura-2•Ca^2+^ complex was taken as 224 nmol/L. Imaging acquisition were done using the software package Meta-Fluor (Molecular Devices).

### Immunohistochemistry

Five-micron thick transverse mouse kidney sections were stained. An anti-Kcne1 (rabbit; 1:200) antibody was used that was kindly provided by Prof. Dr. Richard Warth (University of Regensburg, Germany). Mouse anti-megalin (#75), mouse anti-calbindin (#147), and goat anti-aqp2 (C-17, #6) were all used at 1:200. As secondary antibodies, goat anti-rabbit Alexa 546 (1:300), donkey anti-rabbit Alexa 647 (kindly provided by Prof. Dr. Frank Schweda, University of Regensburg, Germany) (1:300), donkey anti-mouse Alexa 488 (1:300), and donkey anti-goat Cys2 (1:300) were used. Nuclei were stained with Hoechst 33342 (1:100; Aplichem). Immunofluorescence was detected using an Axio Observer microscope equipped with ApoTome.2 and ZEN 2.6 (blue edition) software (Zeiss).

### Live cell staining

HEK293T cells were transfected on glass coverslips with Enhanced Green Fluorescent Protein (eGFP) tagged TMEM16A plasmid. After 48h, cell fluorescence was detected using an Axio Observer microscope equipped with ApoTome.2 and ZEN 2.6 (blue edition) software (Zeiss).

### Semi-quantitative RT-PCR and plasmid generation

Total RNA from HEK293T cells was isolated using NucleoSpin RNA II columns (Macherey-Nagel). Total RNA (0.5 μg/25 μl reaction) was reverse-transcribed using random primers (Promega) and M-MLV Reverse Transcriptase RNase H Minus (Promega). Each RT-PCR reaction contained sense (0.5 μM) and antisense primers (0.5 μM), 0.5 μl cDNA and GoTaq Polymerase (Promega). After 2 min at 95°C, cDNA was amplified (targets 35 cycles, reference GAPDH 25 cycles) for 30 s at 95°C, 30 s at 56°C and 1 min at 72°C. PCR products were visualized by loading on Midori Green Xtra (Nippon Genetics Europe) containing agarose gels and analyzed using Image J 1.52r (NIH). To generate pcDNA31 hKCNE1 and pcDNA31 hKCNE3 plasmids, KCNE1 and KCNE3 were amplified from cDNA derived from 16HBE cells and cloned into NotI/BamHI side of pcDNA31(-)(Invitrogen, Thermo Fisher Scientific). The sequence was verified by sequencing. pcDNA31 hTMEM16A was subcloned into pcDNA31 EGFP to get a C-terminal EGFP tagged fusion protein. hTMEM16A-E727Q point mutation was introduced by site-directed mutagenesis and was verified by sequencing.

### Western blot

Proteins were isolated from HEK293T cells using a sample buffer containing 25 mM Tris–HCl, 150 mM NaCl, 1% Nonidet P-40, 5% glycerol, 1 mM EDTA, and 1% protease inhibitor mixture (Roche). Equal amounts of protein were separated using 8.5% sodium dodecyl sulfate (SDS) polyacrylamide gel. Proteins were transferred to a polyvinylidene difluoride membrane (GE Healthcare) using a semi-dry transfer unit (Bio-Rad). Membranes were incubated with primary anti-Human TMEM16A (rabbit 1:200; Novus biol) antibody for 2.5 h in room temperature. Proteins were visualized using horseradish peroxidase-conjugated secondary antibody and ECL (Thermo Fisher) detection. Beta-Actin was used as a loading control.

### Quantification and statistical analysis

Short-circuit currents (*I*_*sc*_) were analyzed using the Acquire&Analyze II data acquisition system (Physiologic Instruments). Calcium data analysis were performed using the software package Meta-Fluor (Molecular Devices). Fluorescence intensity quantification for live cell staining was done using ImageJ J 1.52r (NIH). Statistical analysis was performed in Excel. Data are reported as mean ± SEM. Student’s *t*-test (for paired or unpaired samples as appropriate) or ANOVA were used. A *p*-value < 0.05 was accepted as significant difference.

## Results

### KCNE1 does not convert TMEM16A into a voltage-dependent Cl^-^ channel, but marginally increases activation of TMEM16A by intracellular Ca^2+^

**Fig. 1:**
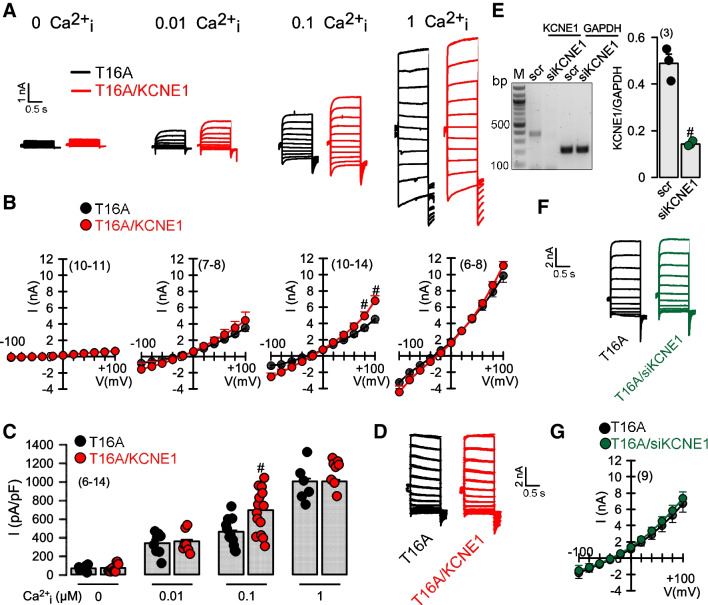
*KCNE1 does not convert TMEM16A into a voltage-dependent Cl*^*-*^ *channel, but slightly enhances activation by intracellular Ca*^*2+*^*.* **A**) Whole cell current overlays obtained from HEK293 cells overexpressing TMEM16A (T16A) or coexpressing T16A and KCNE1, at different (0, 0.01, 0.1, 1 μM) intracellular (pipette) Ca^2+^ concentrations. **B**) Corresponding current/voltage relationships. **C**) Summary of current densities at Vc = +100 mV. **D**) Normalized current overlays showing Mean currents ± SEM from n=8 experiments. **E**) Blot and summary from semiquantitative RT-PCR of KCNE1 and GAPDH, indicating siRNA-knockdown of endogenous KCNE1 expressed in HEK293 cells. **F**) Current overlays of activated TMEM16A currents with or without (siKCNE1) **G**) Corresponding I/V curves. Mean ± SEM (number of experiments). ^#^significant difference when compared to scrambled or T16A (*p*<0.01; unpaired *t*-test)

TMEM16A is a voltage and Ca^2+^ gated Cl^-^ channel [38]. We examined whether coexpression of KCNE1 leads to enhanced activation of TMEM16A by voltage. The abundant human TMEM16A isoform abc (T16A) [14] was expressed in HEK293 cells and whole cell currents were measured in the absence or presence of coexpressed human KCNE1 in the presence of pipette (cytosolic) Ca^2+^ concentrations of 0, 0.01, 0.1, and 1 μM (Fig. 1A). We did not see any activation of T16A by coexpression of KCNE1 at 0 μM Ca^2+^ and in the presence of 5 mM of the Ca^2+^ chelator BABTA-AM. At 0.01 and 0.1 μM Ca^2+^ T16A was partially active in the absence of KCNE1. Coexpression with KCNE1 slightly enhanced Ca^2+^ dependent activation of T16A at 0.1 μM Ca^2+^, but not at lower or maximal (1 μM) Ca^2+^ concentrations (Fig. 1B,C). It should be noted that in contrast to endogenous T16A, overexpressed T16A shows a higher Ca^2+^ sensitivity and is therefore partially active at concentrations of 0.1 μM and even 0.01 μM. This is well observed at the physiological temperature of 37 °C as shown in the present and in previous studies [10, 28, 33].

Normalized T16A overlay mean currents (1 μM Ca^2+^, *n* = 7) did not indicate a change in voltage or time dependence of T16A currents by coexpressed KCNE1 (Fig. 1D). In contrast, KCNE1 conferred time and voltage dependent activation to KCNQ1, as reported earlier [3, 27] (Fig. S1). RT-PCR analysis revealed expression of endogenous KCNE1 in HEK293 cells, which was successfully downregulated by siRNA (Fig. 1E, S9). Nevertheless, knockdown of endogenous KCNE1 had no effect on T16A currents (Fig. 1F,G). Moreover, we expressed the T16A splice variant ab (T16A_ab_), which had been reported to show a larger instantaneous (non-voltage) dependent current even at low intracellular Ca^2+^ [14]. However, coexpression of KCNE1 did also not confer voltage- or time- dependence to T16Aab (Fig. S2).

### KCNE1 marginally enhances activation of TMEM16A by purinergic stimulation

**Fig. 2:**
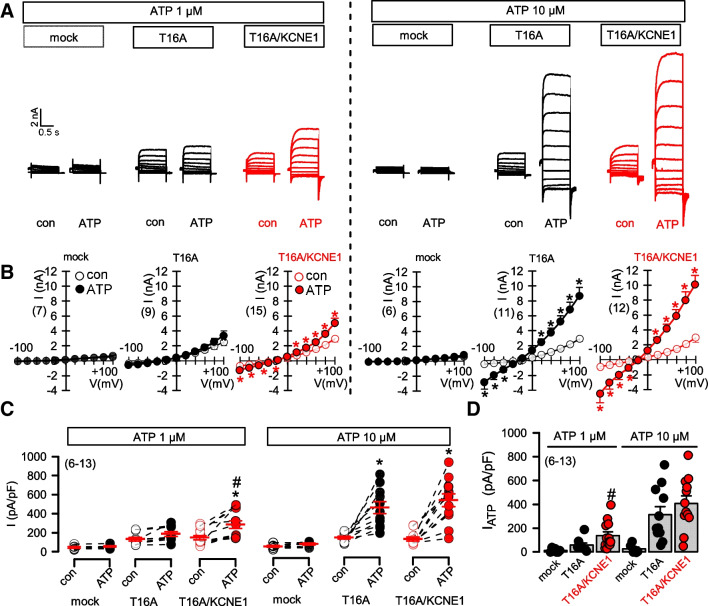
*KCNE1 slightly enhances activation of TMEM16A by purinergic stimulation.* **A**) T16A whole cell currents activated by ATP (1 μM, left and 10 μM, right) in mock-transfected HEK293 cells and cells expressing T16A or coexpressing T16A and KCNE1. **B**) Corresponding current/voltage relationships. **C**) Summary of current densities at Vc = +100 mV. **D**) Summary of ATP-activated current densities. Mean ± SEM (number of experiments). *significant increase by ATP (*p*<0.001; paired *t*-test). ^#^significant difference when compared to T16A (*p*<0.01; unpaired *t*-test)

T16A is activated by increase in cytosolic Ca^2+^ through stimulation of purinergic receptors. While 1 μM ATP was not sufficient to activate T16A alone, activation of T16A was observed in cells coexpressing KCNE1 (Fig. 2 AB, left panels, C,D). However, KCNE1 did not affect pronounced activation of T16A at 10 μM ATP (Fig. 2 AB, right panels, C,D).

**Fig. 3:**
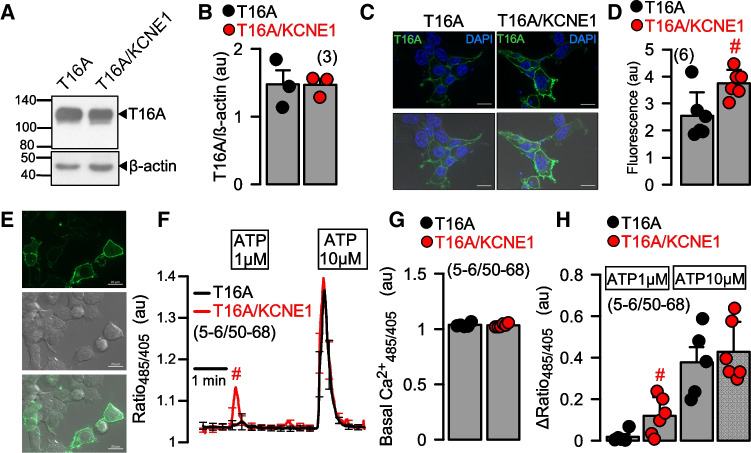
KCNE1 enhances membrane expression of TMEM16A and intracellular Ca^2+^ signals. **A, B)** Western blot showing expression of T16A in the absence or presence KCNE1. **C, D)** Immunocytochemistry showing expression of T16A in HEK293 cells in the absence or presence of coexpressed KCNE1. **E)** Fluorescence of plasma membrane localized Ca^2+^ sensor Pl-G-CaMP2. **F)** Ca^2+^ increase near plasma membrane by ATP, as detected by PI-G-CaMP2. **G, H)** Basal Ca^2+^ and ATP-induced Ca^2+^ increase in the absence or presence of KCNE1. Mean ± SEM (number of experiments). ^#^significant difference when compared to T16A (*p*<0.01; unpaired *t*-test)

The results show again the slight increase in Ca^2+^ -dependent activation of T16A in the presence of KCNE1. KCNE1 did not affect total expression of T16A, as detected by Western blotting (Fig. 3A,B, S9). However, KCNE1 slightly enhanced plasma membrane localization of T16A (Fig. 3C,D). Notably, it was shown earlier that KCNE1 augments membrane localization of KCNQ1 [15].

KCNE1 might increase intracellular Ca^2+^ and thereby affect expression of ion channels in the plasma membrane. To explore intracellular Ca^2+^ levels near the plasma membrane, we used the membrane-bound Ca^2+^ sensor Pl-G-CaMP2. Basal sub-membranous Ca^2+^ was not affected by coexpression of KCNE1, but stimulation with a low concentration of ATP (1 μM) induced a small but significant rise in intracellular Ca^2+^ that was not observed in the absence of KCNE1 (Fig. 3E–H). This effect of KCNE1 on ATP-induced increase of [Ca^2+^]_i_ is probably due to enhanced membrane expression of TMEM16A. TMEM16A is known to augment inositol trisphosphate (IP_3_)-induced Ca^2+^ increase [6, 18].

### KCNE1 slightly enhances Ca^2+^-dependent activation of the low Ca^2+^-affinity mutant TMEM16A-E727Q

In contrast to Ávalos Prado et al., we did not detect additional voltage- or time-dependence of T16A by coexpression with KCNE1. In all experimental conditions T16A required Ca^2+^ to be activated. We expressed T16A-E727Q, a mutant with strongly reduced Ca^2+^ affinity [34]. This mutant was not activated by 1 or 10 μM ATP and coexpression with KCNE1 had no effects (Fig. S3A,B). However, a large rise in [Ca^2+^]_i_ by 1 or 5 μM ionomycin activated T16A-E727Q and activation by 1 μM ionomycin was slightly augmented in the presence of KCNE1 (Fig. S3C,D). Thus, for T16A-E727Q the small effect of KCNE1 is shifted to higher Ca^2+^ concentration range. This again demonstrates that i) the presence of Ca^2+^ is required for KCNE1 to exert its effect in T16A, ii) the effect of KCNE1 is independent of IP_3_, and iii) most likely the marginal effect of KCNE1 is explained by a slight increase in membrane expression of T16A induced by KCNE1.

### N-terminal KCNE1 peptide enhances ATP-dependent activation of overexpressed and endogenous TMEM16A probably by a transient increase in intracellular Ca^2+^

**Fig. 4:**
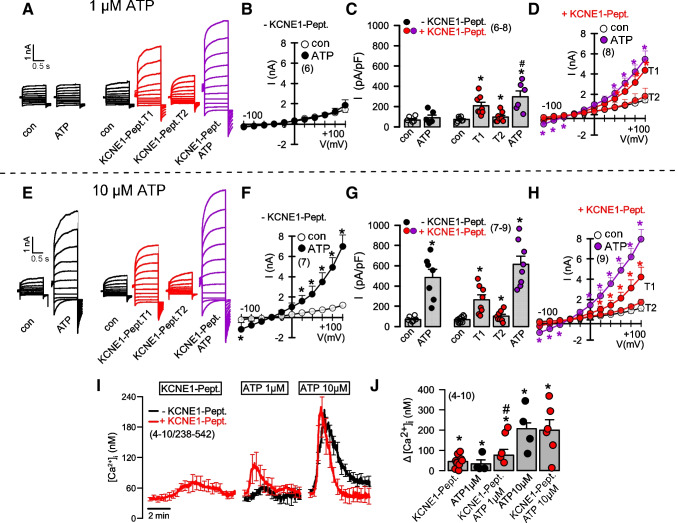
A KCNE1 peptide enhances activation of TMEM16A by ATP*.* **A**) Whole cell current overlays obtained from HEK293 cells overexpressing T16A before and after stimulation with 1 μM ATP. Application of N-terminal KCNE1 peptide (KCNE1-Pept., 100 μM) acutely activated T16A (time point T1). Activation was transient (T1) and collapsed within 1 min (time point T2), while subsequent activation by ATP was largely augmented. **B–D**) Corresponding current/voltage relationships in the absence and presence of KCNE1-Pept. and summary of current densities at Vc = +100 mV. **E**) Whole cell currents activated 10 μM ATP in the absence and presence of KCNE1-Pept.. **F–H**) Corresponding current/voltage relationships and summary of current densities. **I, J**) Summary traces of intracellular Ca^2+^ showing increase by KCNE1-Pept. and ATP-induced increase in the absence or presence of KCNE1-Pept. Mean ± SEM (number of experiments). *significant activation by ATP or KCNE1-Pept., respectively (*p*<0.01; paired *t*-test). ^#^significant difference when compared to absence of KCNE1-Pept. (*p*<0.01; unpaired *t*-test)

Ávalos Prado et al. report a peptide of 13 residues preceding the transmembrane domain of KCNE1 (Nter13) that is sufficient to activate T16A. This observation was related to a previous study that showed activation of an unknown endogenous Cl^-^ current by KCNE1 peptides in *Xenopus* oocytes [1, 2]. We therefore examined the effects of this peptide (here called KCNE1-Pept.) on T16A. As shown in Fig. 4, application of this KCNE1-Pept. activated T16A, but activation (measured at time point T1) was transient and quickly collapsed within 10 s (measured at time point T2). Subsequent stimulation with 1 μM ATP again reactivated T16A (Fig. 4A–D). In the absence of KCNE1-Pept. 1 μM ATP was unable to activate T16A. Like KCNE1 also KCNE1-Pept. seems to increase intracellular Ca^2+^ and/or may somehow sensitize T16A towards stimulation by Ca^2+^. At 10 μM ATP, KCNE1-Pept. no longer had an effect on activation of T16A (Fig. 4E–H). Indeed, application of KCNE1-Pept. slightly and transiently enhanced intracellular Ca^2+^ and augmented Ca^2+^ increase by 1 μM, but not by 10 μM ATP (Fig. 4I,J). Under no circumstances KCNE1-Pept. induced voltage dependence of T16A. Finally, KCNE1-Pept., did not at all affect activation of KCNQ1 (Fig. S4). The data strongly suggest a non-specific effect of KCNE1-Pept. and coexpressed KCNE1 on intracellular Ca^2+^ which affects activation of T16A, but which is unrelated to a specific effect of KCNE1-Pept./ KCNE1 on T16A. These findings may be explained by the fact that Orai1 Ca^2+^ influx channels are expressed endogenously in HEK cells and have been reported to be activated by voltage [31]. As shown below, positive charges within KCNE1 or KCNE3 indeed induce a nonspecific Ca^2+^ influx (transient for KCNE1-Pept.; more permanent for coexpressed KCNE1).

**Fig. 5:**
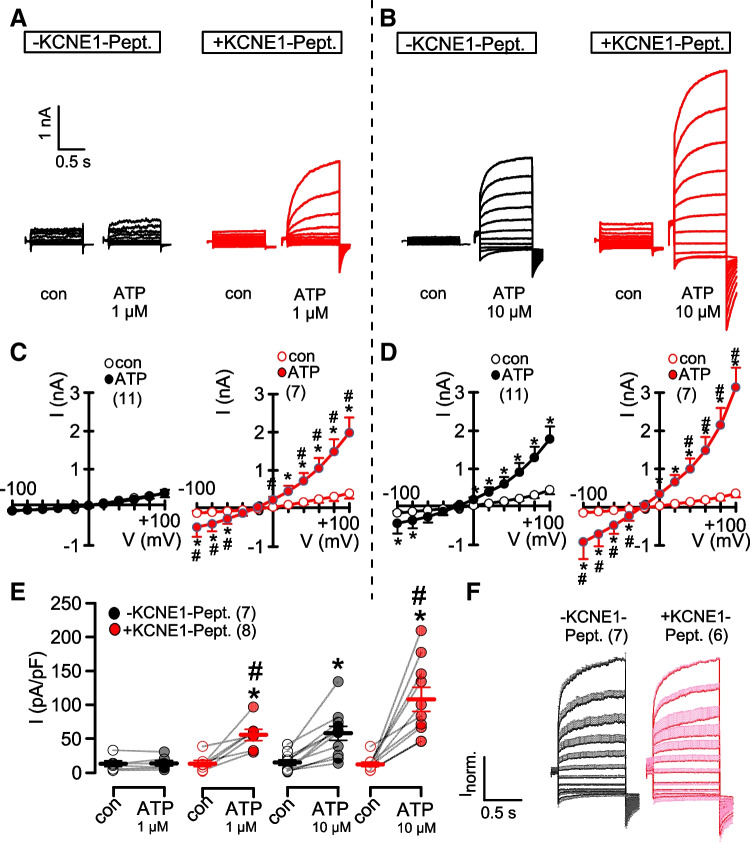
A KCNE1 peptide enhances activation of endogenous TMEM16A by ATP*.* **A, B**) Whole cell current overlays obtained from human airway CFBE cells expressing endogenous T16A. Cells were stimulated with 1 or 10 μM ATP, in the absence or presence of KCNE1-Pept. (100 μM). **C, D**) Corresponding current/voltage relationships. **E**) Summary of current densities at Vc = +100 mV. **F**) Normalized T16A current overlays in the absence of presence of KCNE1-Pept. indicating no change in time-dependent activation or voltage dependence of T16A by KCNE1-Pept.. Mean ± SEM (number of experiments). *significant activation by ATP (*p*<0.01; paired *t*-test). ^#^significant difference when compared to absence of KCNE1-Pept. (*p*<0.05; unpaired *t*-test)

Overexpressed and endogenous TMEM16A respond somewhat different concerning Ca^2+^ sensitivity, activation by Ca^2+^ increase or inhibition by small molecules [9, 28, 33]. We therefore examined the effect of KCNE1-Pept. on T16A in CFBE epithelial cells, which express high levels of endogenous TMEM16A [7]. Notably, endogenous TMEM16A was not activated by KCNE1-Pept., but activation by ATP (1 or 10 μM) was strongly augmented in the presence of the peptide (Fig. 5A–E). Again, KCNE1-Pept. did not affect voltage dependence or time dependence of T16A (Fig. 5F).

### KCNE1 is not expressed in mouse RPT cells and therefore does not affect Ca^2+^ signaling or activation of TMEM16A by AngII or ATP

**Fig. 6:**
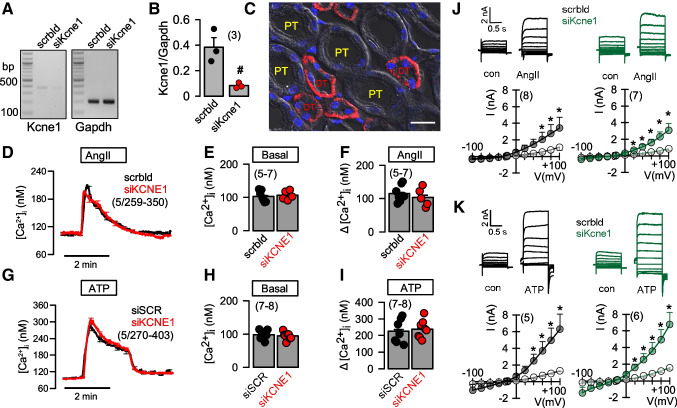
KCNE1 is not expressed in primary proximal tubular epithelial cells and does not affect activation of T16A by AngII or ATP. **A, B**) Semiquantitative RT-PCR indicating insignificant expression of mRNA for KCNE1 in primary proximal tubular epithelial cells, which nevertheless could be knocked-down by siRNA. scrbld indicates treatment by scrambled RNA. **C**) Immunocytochemistry showing expression of KCNE1 in the basolateral membrane of renal distal tubular (DT) epithelial cells but not in the proximal tubule (PT). **D**) Summary tracings showing an increase in intracellular Ca^2+^ by stimulation with AngII (100 μM). **E, F**) Summaries for basal and AngII-induced Ca^2+^ rise. Knockdown of KCNE1-expression had no effect on intracellular Ca^2+^ signals. **G–I**) Summary tracings and summary bar graphs showing basal Ca^2+^ and ATP (100 μM)-induced increase in intracellular Ca^2+^. **J, K**) Activation of T16A whole cell currents by AngII (100 μM) or ATP (100 μM) in primary proximal tubular epithelial cells treated with scrambled (scrbld) RNA or siRNA-KCNE1. Mean ± SEM (number of experiments). *significant activation by AngII or ATP (*p*<0.01; paired *t*-test). ^#^significant difference when compared to scrambled (*p*<0.05; unpaired *t*-test)

In their paper, Ávalos Prado et al discuss a physiological role of the KCNE1-TMEM16A complex for water reabsorption in the proximal tubule [2]. We have analyzed mRNA for Kcne1 in isolated mouse renal proximal tubular epithelial (RPT) cells, but could only detect a negligible expression of Kcne1-mRNA. Nevertheless, the minimal expression of Kcne1-mRNA could be downregulated by siRNA (siKcne1) (Fig. 6A,B, S9). Proximal tubules can be easily discriminated from other tubular section such as distal tubule and collecting ducts due to a pronounced brush boarder, which is clearly visible in Fig. 6, and Fig. S5. Moreover, we included immunocytochemistry of Kcne1 co-stained with the tubular marker proteins megalin (proximal tubule), calbindin (distal tubule), and aquaporin 2 (Aqp2; collecting duct). Immunocytochemistry clearly indicated expression of Kcne1 in the basolateral membrane of distal tubule, but not in proximal tubule or collecting duct (Fig. S5).

We analyzed intracellular Ca^2+^ signals elicited by stimulation of isolated RPT cells with either AngII or ATP. Increase in both peak and plateau Ca^2+^ was not affected by knockdown of Kcne1, probably due to the negligible expression of Kcne1 (Fig. 6D–I). Ávalos Prado et al reported loss of AngII-activated whole cell currents in RPT cells from Kcne1-/- mice. In contrast, our data do not support a role of KCNE1 for activation of Tmem16A in RPT cells, as knockdown of Kcne1 did not affect activation by AngII or ATP (Fig. 6J,K). This is not surprising given the lack of expression of Kcne1 in the proximal tubule. Taken together, we find no substantial expression of Kcne1 in the apical membrane of mouse proximal tubule and no evidence for a role of Kcne1 in regulating TMEM16A. TMEM16A in renal proximal tubule is activated by AngII and ATP which both increase intracellular Ca^2+^. TMEM16A whole cell currents are abolished in Tmem16a-/- mice as shown in our previous report [13], but are unaffected by siRNA-Kcne1.

### Positively charged poly-L-lysine activates TMEM16A

**Fig. 7:**
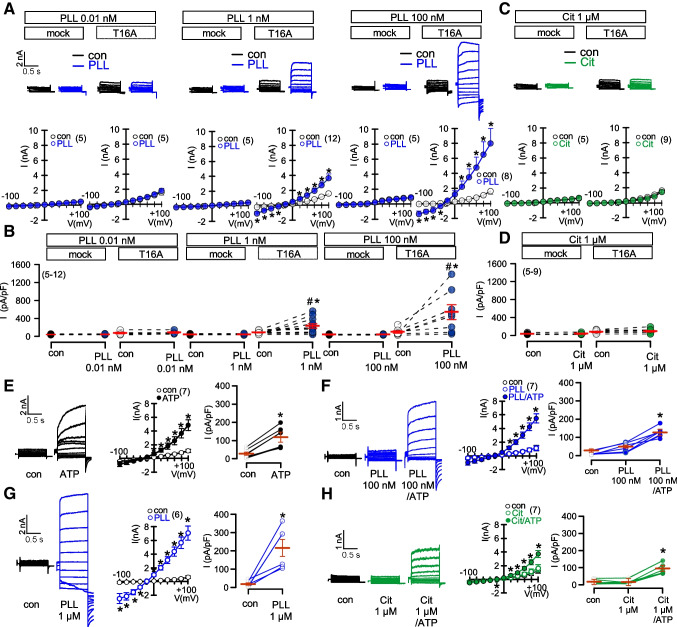
*Extracellular application of poly-L-lysine supports Ca*^*2+*^*-dependent activation of TMEM16A.* **A**) Whole cell currents activated by extracellular poly-L-Lysine (PLL; 0.01, 1, 100 nM) in mock-transfected and T16A-overexpressing HEK293 cells. Overlay currents and corresponding I/V curves. **B**) Calculated current densities. **C,D**) Whole cell currents and current densities in the absence or presence of 1 μM citrate. **E, F**) Endogenous T16A whole cell currents activated by ATP (100 μM) in CFBE cells in the absence or presence of 100 nM poly-L-Lysine. **G**) Activation of T16A by 1 μM PLL. **H**) Whole cell currents and current densities activated by ATP in the presence of 1 μM citrate. Mean ± SEM (number of experiments). *significant activation by ATP and PLL, respectively (*p*<0.05; paired *t*-test). ^#^significant difference when compared to mock (*p*<0.05; unpaired *t*-test)

KCNE1 and KCNE1-pept. do not confer voltage dependence to T16A, but slightly increase intracellular Ca^2+^ and activate T16A. We asked whether this could be due to a non-specific effect exerted by positive charges located in the extracellular N-terminus of KCNE1 and KCNE1-pept. (Fig. S7A). To test this hypothesis, we applied low concentrations of polycationic poly-L-lysine (PLL) to the extracellular bath solution and indeed found a pronounced activation of T16A. No currents were activated in mock transfected HEK293 cells (Fig. 7A,B). Notably, negatively charged citrate did not activate T16A (Fig. 7C,D). Moreover, similar to citrate also hyaluronic acid (100 nM) had only a variable and small effect on TMEM16A activity (ΔI = 62 ± 30 pA/pF; *n*=5). We also examined the effects on endogenous T16A expressed in CFBE cells. 100 nM PLL augmented ATP-induced T16A currents (Fig. 7E,F), while 1 μM PLL itself strongly activated T16A (Fig. 7G). Again, no significant effects were seen with citrate (Fig. 7H). Similar to KCNE1-pept., also PLL increased intracellular Ca^2+^ which is probably the cause for activation of T16A (Fig. S6A,B). In fact, when Ca^2+^ was removed from the extracellular bath solution (0 Ca^2+^ + 5 mM Ca^2+^ chelator BAPTA-AM), no activation was observed even at 1 μM PLL. Taken together, the K^+^ channel ß-subunit KCNE1 does not shift TMEM16A from a Ca^2+^ dependent to a voltage dependent Cl^-^ channel. Under all conditions examined here, the presence of cytosolic Ca^2+^ is indispensable for activation of TMEM16A. KCNE1 is not expressed in renal proximal tubules and does not control the activity of TMEM16A in RPT cells.

## Discussion

### TMEM16A is a Cl^-^ channel gated by depolarized voltages and Ca^2+^ but not by KCNE1

According to the paper by Ávalos Prado et al, KCNE1 interacts with TMEM16A and confers voltage-dependent activation to TMEM16A without the need to increase intracellular Ca^2+^ [2]. However, TMEM16A per se is a voltage-dependent Cl^-^ channel, as Ca^2+^ binding occurs in a voltage dependent manner, i.e., binding of Ca^2+^ is facilitated at depolarized membrane voltages [24, 36, 38]. Unlike described in the paper by Ávalos Prado et al, we detected no increase in voltage dependence of T16A, either by coexpression of KCNE1 nor by an extracellular KCNE1 peptide. In contrast, KCNE1 clearly induced a typical time-dependent activation of KCNQ1 (Fig. S1).

### KCNE1 and poly-L-lysine augment ATP-induced Ca^2+^ increase and directly increase intracellular Ca^2+^

KCNE1 and KCNE-Pept. augmented Ca^2+^ increase induced by ATP, and enhanced activation of T16A (Figs. 2–5). We speculate that the positive charges in the extracellular N-terminus of KCNE1 may impose a moderately positive potential, which may affect receptor-mediated ER-store release or Ca^2+^ influx [16]. As an example, polycation-dextran induces migration of macrophages by increasing ER-store release and Ca^2+^ influx [12]. KCNE3 is another ß-subunit of KCNQ1 that turns KCNQ1 into a voltage-independent channel [4]. Similar to KCNE1, it also carries positive charges in the extracellular N-terminus (Fig. S7). We found that similar to KCNE1 also KCNE3 slightly augments ATP-activation of T16A without changing the voltage dependence of T16A (Fig. S7).

We also applied KCNE1-Pept. to epithelial monolayers mounted in an Ussing chamber, which induced a pronounced charge artefact that led to an offset current, supporting the idea of a charge artefact underlying the effects of KCNE1 (Fig. S8). Large potential changes are induced by polycationic poly-L-lysine, causing a substantial Ca^2+^ increase and direct activation of T16A (Figs. S6, S7). Notably, an earlier study reported poly-L-lysine induced release of prostaglandin E2 and Ca^2+^ increase in mesangial cells [26]. Ávalos Prado et al found that the voltage-insensitive T16A mutant 444EEEE447/444AAAA447 [38] did not prevent voltage-activation by KCNE1 [2]. However, in our study the natural T16A splice variant TMEM16A_ab_ that showed a largely attenuated voltage dependence [14], was not voltage activated by coexpression of KCNE1 (Fig. S2). Taken together we do not find evidence for a specific functional regulation of T16A by KCNE1.

### Insignificant expression of KCNE1 in mouse proximal tubular epithelial cells

TMEM16A is expressed in the apical membrane of renal tubular epithelial cells [13]. It contributes essentially to autosomal dominant polycystic kidney disease [5, 8]. Sugimoto et al and Vallon et al provided some evidence for expression of KCNE1 in proximal tubule of rat [32] and mouse [37], while Ávalos Prado et al do not show any expression data [2]. In contrast to previous studies, cell-type specific analysis of mRNA in mouse kidney demonstrated expression of KCNE1 in distal but not proximal tubule (https://cello.shinyapps.io/kidneycellexplorer/). This is in full agreement with our present data demonstrating (by RT-PCR and immunohistochemistry) the absence of relevant expression of KCNE1 in proximal tubule, but indicating clear expression in the basolateral membrane of distal tubule (Fig. 6, Fig. S5).

According to the data presented by Ávalos Prado et al, T16A whole cell currents were not activated by AngII in RPT cells from KCNE1-/- mice. This is rather surprising given the fact that AngII increases intracellular Ca^2+^, which should be able to activate T16A, even in the absence of KCNE1. In clear contrast to their finding, we report that siRNA for KCNE1 does not change Ca^2+^-dependent activation of overexpressed or endogenous T16A in HEK293 cells, or in primary mouse RPT cells (Figs. 1,6). In summary, our data do not provide evidence for a physiological regulation of TMEM16A by KCNE1.

## Supplementary Information

ESM 1

## Data Availability

All original data are available on request.
