# Supplementary material for: KCNE1 does not shift TMEM16A from a Ca2+ dependent to a voltage dependent Cl- channel and is not expressed in renal proximal tubule
Source: Pflugers Arch. 2023 Jul 13;475(8):995–1007. doi: 10.1007/s00424-023-02829-5 (PMC10359377; doi:10.1007/s00424-023-02829-5)
Supplement: Supplementary file 1 — ESM 1 [file 424_2023_2829_MOESM1_ESM.zip › FigS1.pdf]

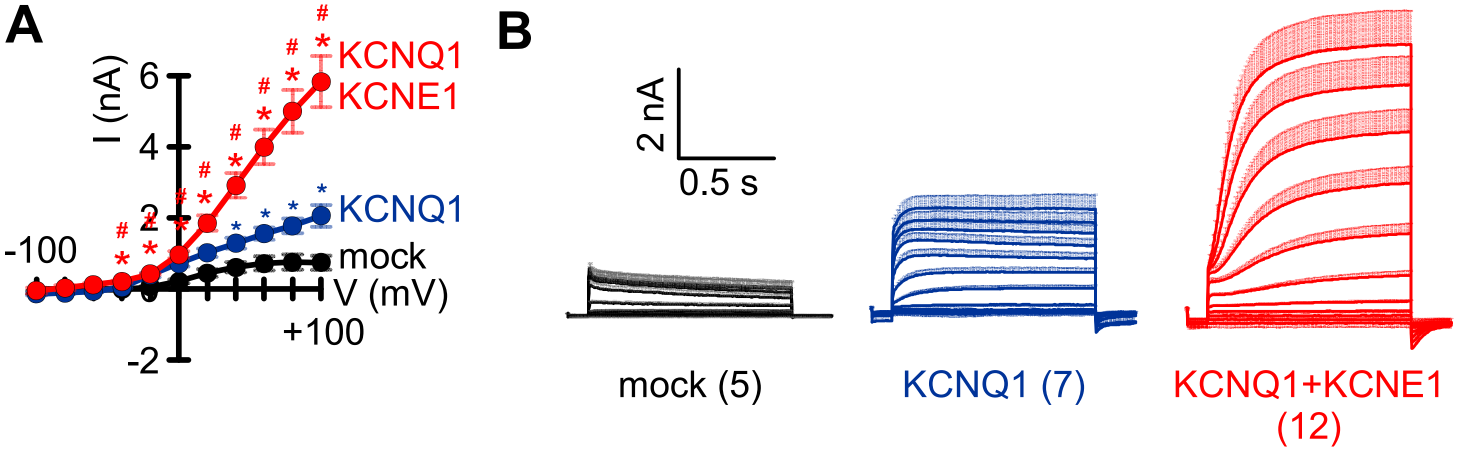

Supplementary Figure 1. *Change of time- and voltage-dependence of KCNQ1 currents by coexpression of KCNE1.* **A)** Whole cell summary currents detected in mock-transfected, hKCNQ1, and hKCNQ1/hKCNE1-expressing HEK293 cells. **B)** Corresponding current/voltage relationships. Mean ± SEM (number of experiments). *significant difference to mock ($p<0.05$; unpaired t-test). #significant difference to KCNQ1 ($p<0.05$; unpaired t-test).
