# Supplementary material for: KCNE1 does not shift TMEM16A from a Ca2+ dependent to a voltage dependent Cl- channel and is not expressed in renal proximal tubule
Source: Pflugers Arch. 2023 Jul 13;475(8):995–1007. doi: 10.1007/s00424-023-02829-5 (PMC10359377; doi:10.1007/s00424-023-02829-5)
Supplement: Supplementary file 1 — ESM 1 [file 424_2023_2829_MOESM1_ESM.zip › FigS2.pdf]

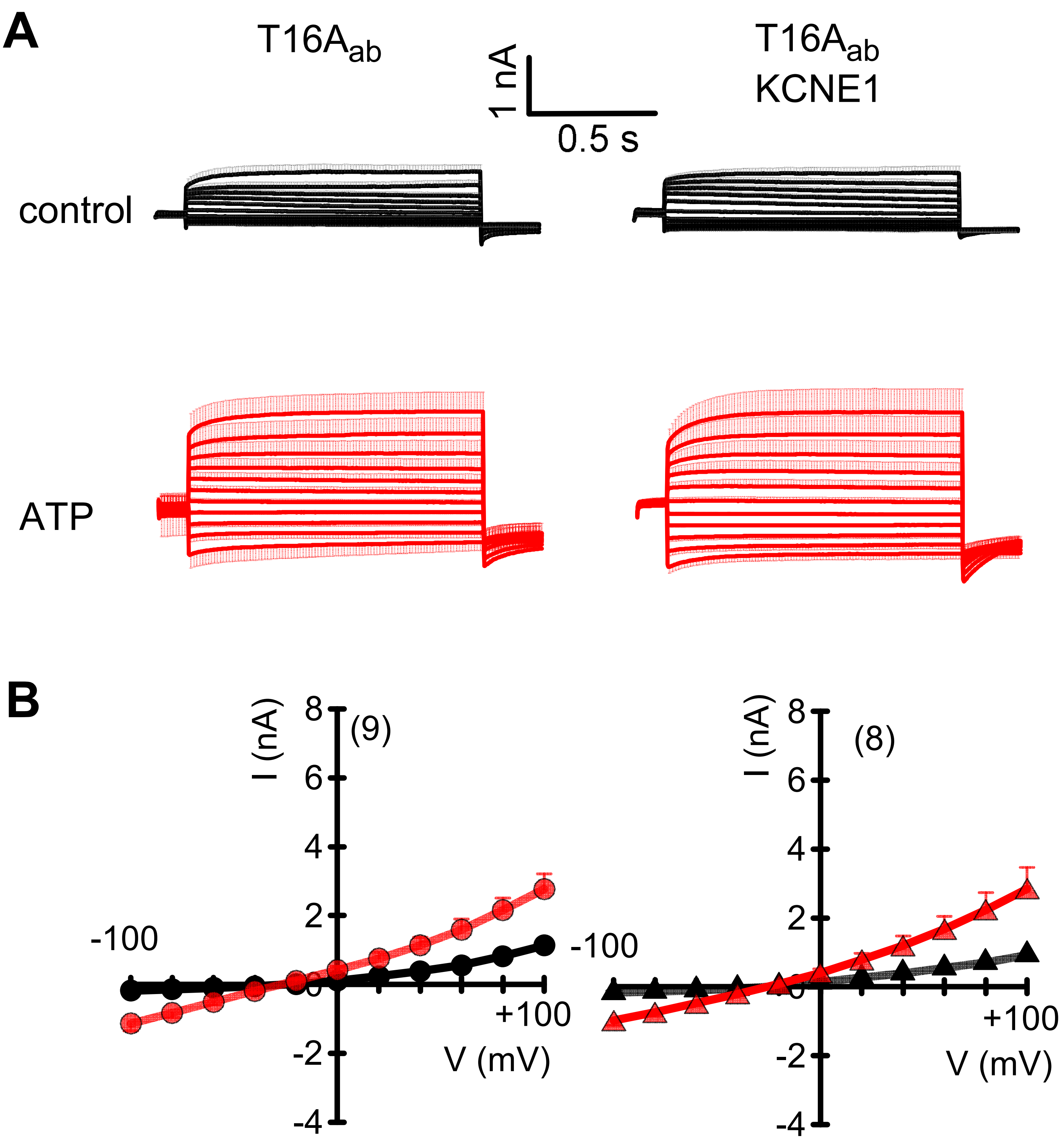

Supplementary Figure 2. No *Change of time- and voltage-dependence of the TMEM16A$_{ab}$ variant by coexpression of KCNE1.* **A)** Whole cell summary current overlays under control after activation by 1 µM ATP in HEK293 cells expressing T16A$_{ab}$ variant (left) or coexpressing T16A$_{ab}$ and KCNE1 (right). **B)** Corresponding current/voltage relationships. Mean ± SEM (number of experiments). *significant activation by ATP (paired t-test).
