# Supplementary material for: KCNE1 does not shift TMEM16A from a Ca2+ dependent to a voltage dependent Cl- channel and is not expressed in renal proximal tubule
Source: Pflugers Arch. 2023 Jul 13;475(8):995–1007. doi: 10.1007/s00424-023-02829-5 (PMC10359377; doi:10.1007/s00424-023-02829-5)
Supplement: Supplementary file 1 — ESM 1 [file 424_2023_2829_MOESM1_ESM.zip › FigS3.pdf]

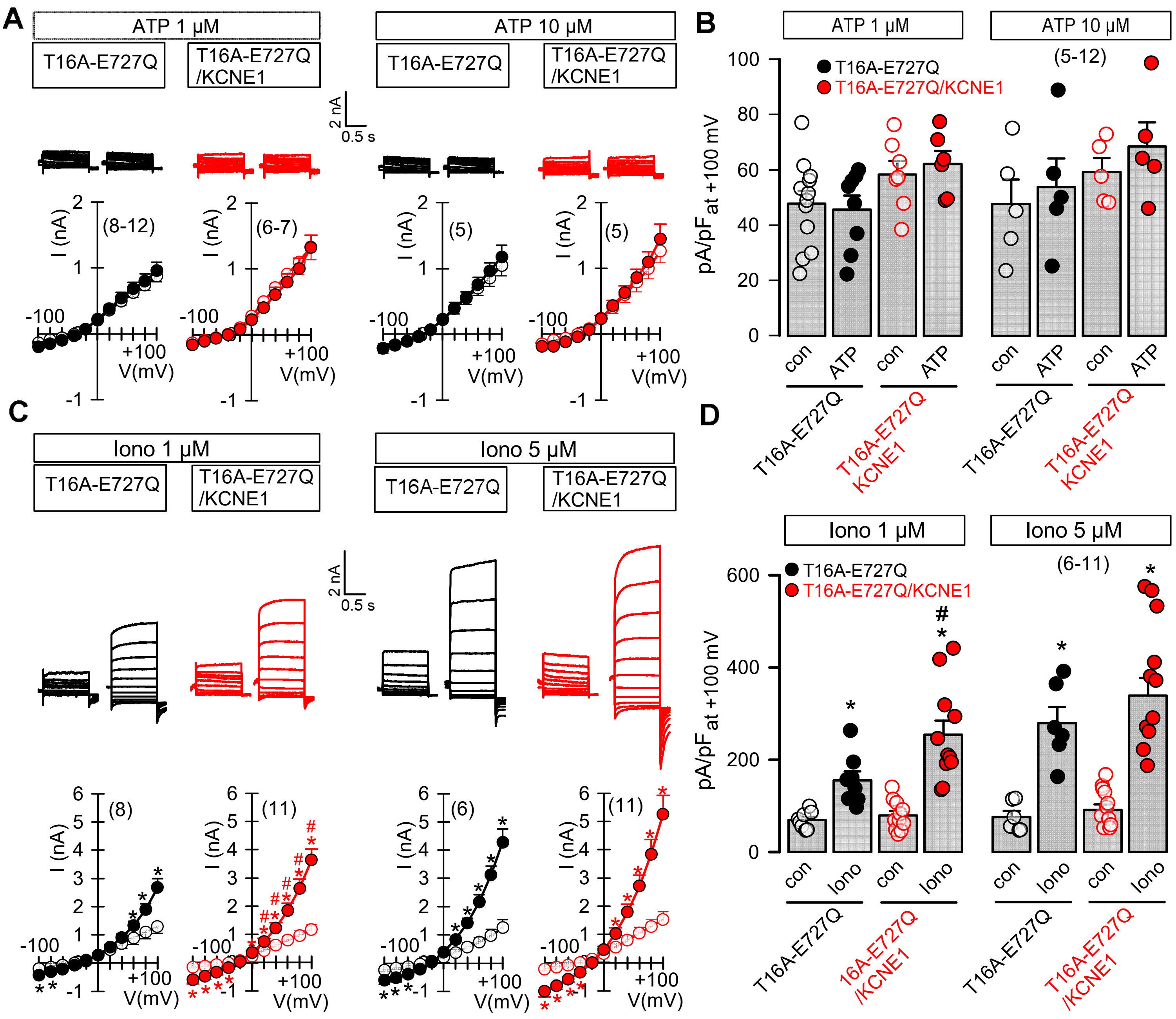

Supplementary Figure 3. *A TMEM16A mutant with reduced Ca^{2+} sensitivity shows enhanced Ca^{2+}-dependent activation when coexpressed with KCNE1.* **A,B**) Lack of activation of T16A-E727Q whole cell currents by 100 µM ATP, independent of coexpression of KCNE1. **C,D**) Activation of T16A-E727Q whole cell currents by 1 and 5 µM ionomycin. At 1 µM ionomycin, activation of T16A-E727Q is significantly enhanced in the presence of coexpressed KCNE1. Mean ± SEM (number of experiments). *significant increase by ionomycin ($p<0.05$; paired t-test). #significant difference when compared to T16A-E727Q ($p<0.01$; unpaired t-test).
