# Supplementary material for: KCNE1 does not shift TMEM16A from a Ca2+ dependent to a voltage dependent Cl- channel and is not expressed in renal proximal tubule
Source: Pflugers Arch. 2023 Jul 13;475(8):995–1007. doi: 10.1007/s00424-023-02829-5 (PMC10359377; doi:10.1007/s00424-023-02829-5)
Supplement: Supplementary file 1 — ESM 1 [file 424_2023_2829_MOESM1_ESM.zip › FigS4.pdf]

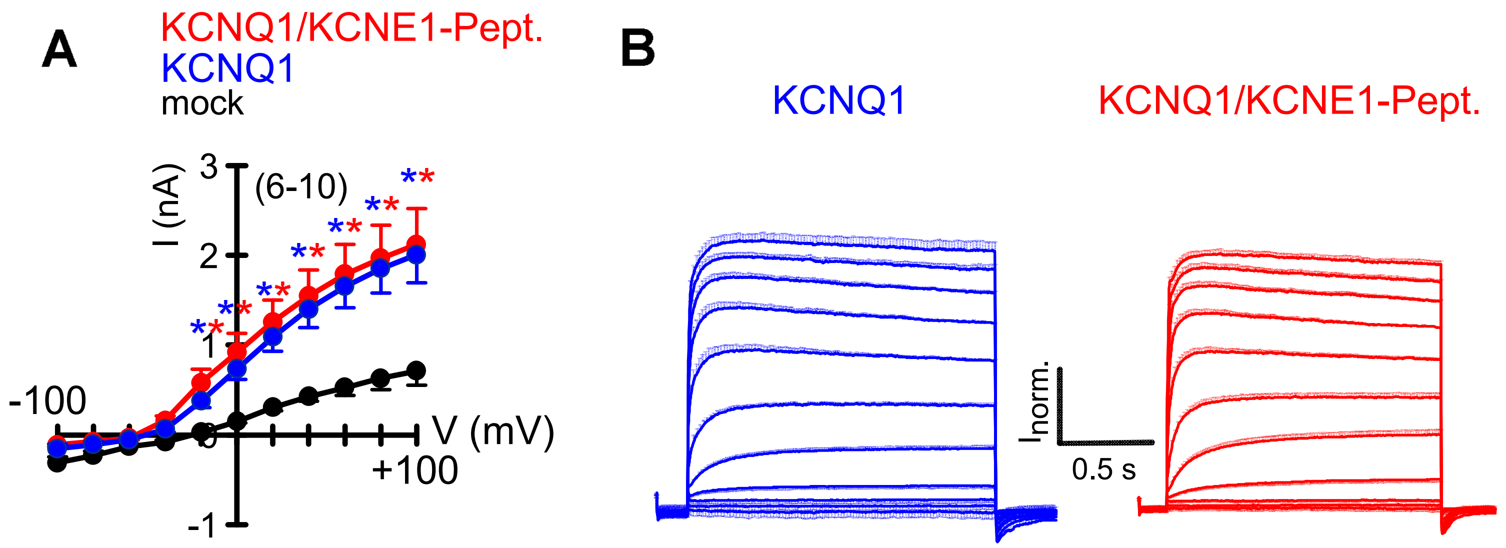

Supplementary Figure 4. *KCNE1-Pept. does not change KCNQ1 currents.* **A,B**) Current/voltage relationships and current overlays obtained from HEK293 cells overexpressing KCNQ1 or mock transfected cells. Application of KCNE1-Pept. did not affect time dependence or voltage dependence of KCNQ1. Mean ± SEM (number of experiments). *indicates significant difference when compared to mock ($p<0.01$; unpaired t-test).
