# Supplementary material for: KCNE1 does not shift TMEM16A from a Ca2+ dependent to a voltage dependent Cl- channel and is not expressed in renal proximal tubule
Source: Pflugers Arch. 2023 Jul 13;475(8):995–1007. doi: 10.1007/s00424-023-02829-5 (PMC10359377; doi:10.1007/s00424-023-02829-5)
Supplement: Supplementary file 1 — ESM 1 [file 424_2023_2829_MOESM1_ESM.zip › FigS5.pdf]

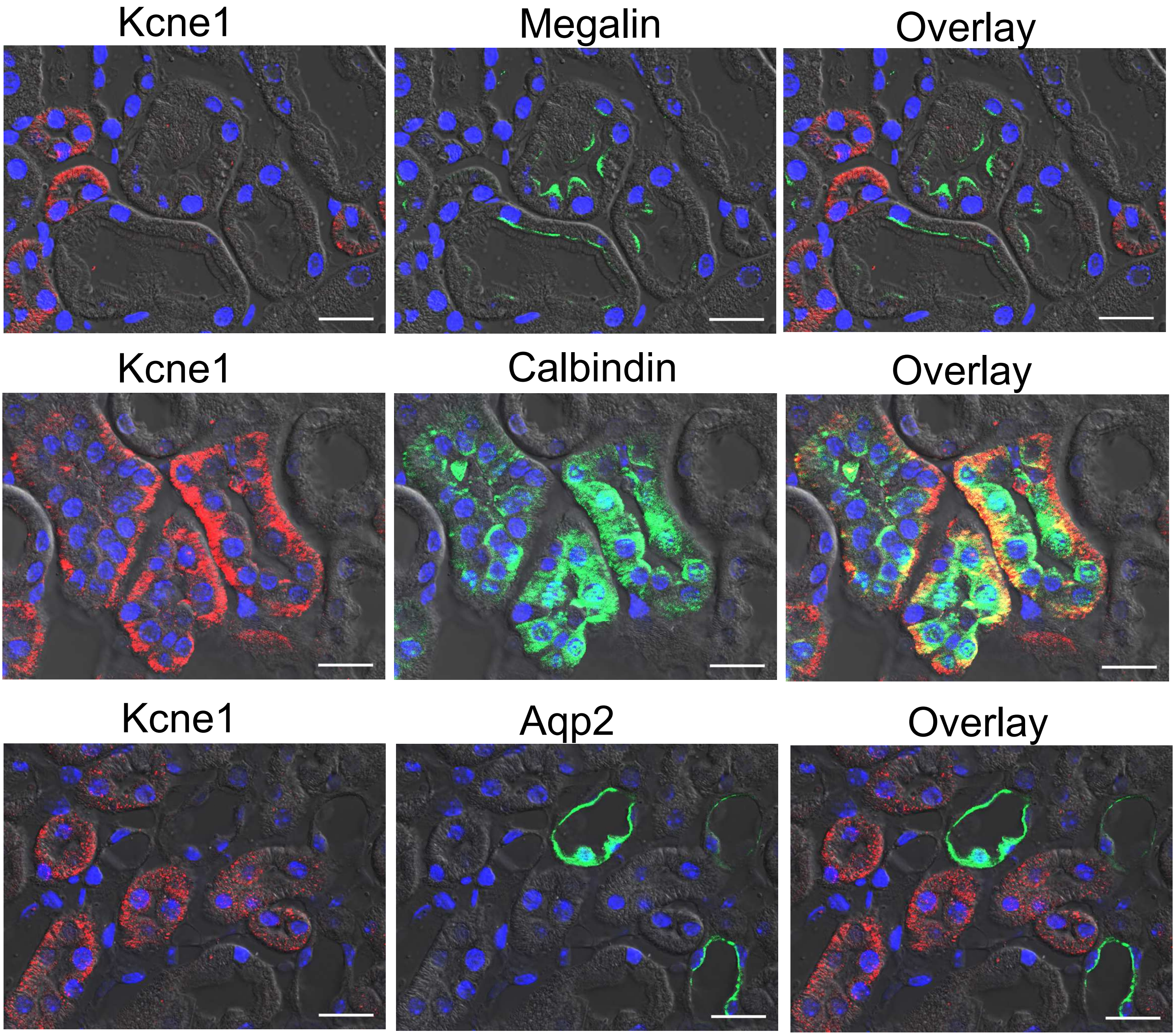

Supplementary Figure 5. *Basolateral staining of Kcne1 in mouse distal but not proximal tubular epithelial cells.* Kcne1 (red) is detected in the basolateral membrane of distal tubule, where also calbindin is expressed. Megalin and Aqp2 were stained to mark proximal tubule and collecting duct, respectively. DAPI staining of nuclei in blue. 63x magnification, bars = 20 μm.
