# Supplementary material for: KCNE1 does not shift TMEM16A from a Ca2+ dependent to a voltage dependent Cl- channel and is not expressed in renal proximal tubule
Source: Pflugers Arch. 2023 Jul 13;475(8):995–1007. doi: 10.1007/s00424-023-02829-5 (PMC10359377; doi:10.1007/s00424-023-02829-5)
Supplement: Supplementary file 1 — ESM 1 [file 424_2023_2829_MOESM1_ESM.zip › FigS6.pdf]

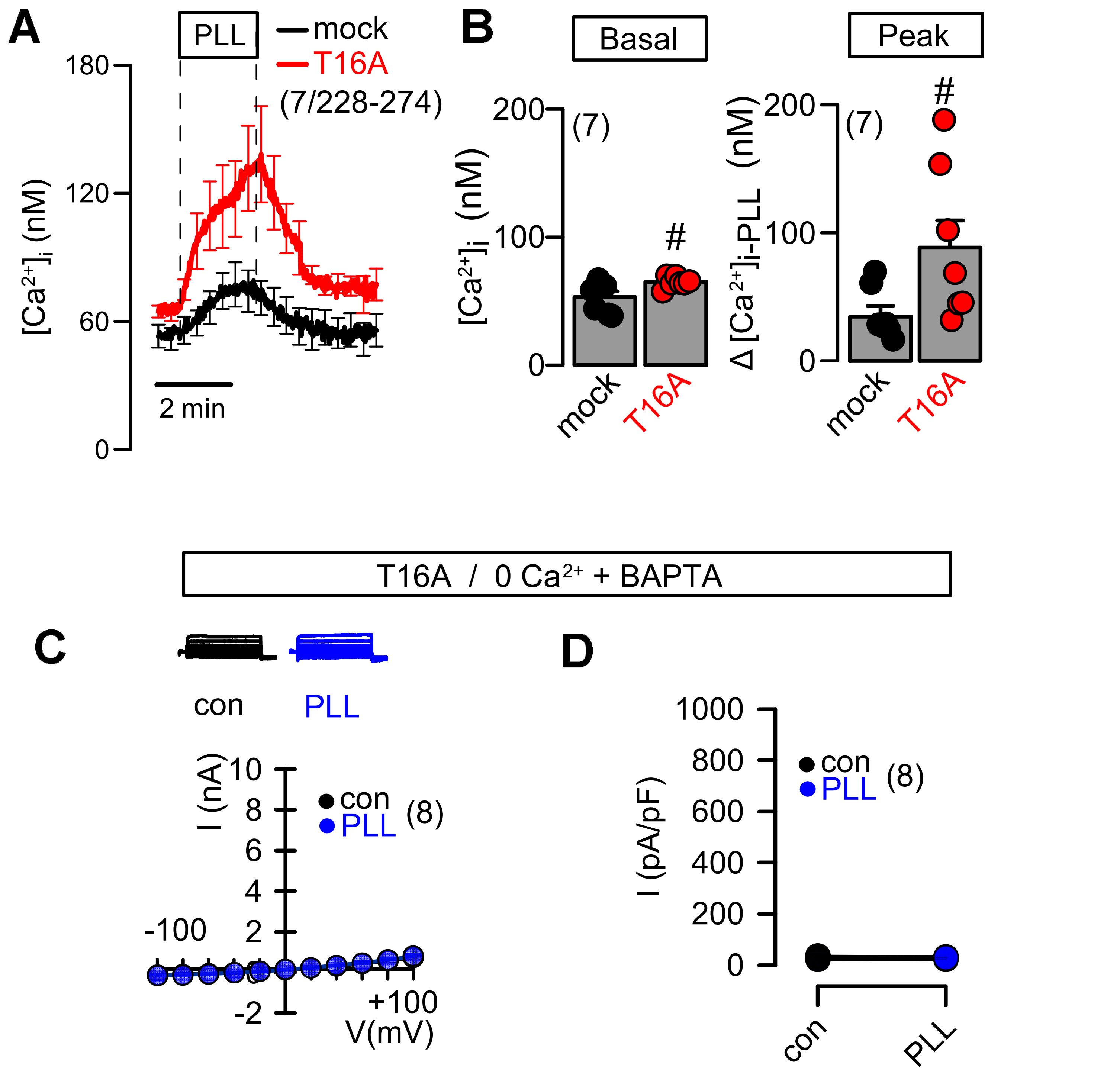

Supplementary Figure 6. *PLL increases cytosolic Ca^{2+} and activates TMEM16A, but not in the absence of Ca^{2+}.* **A)** Intracellular Ca^{2+} assessed by Fura2 in mock transfected HEK293 cells and cells overexpressing T16A. PLL (10 nM) enhanced intracellular Ca^{2+}. Ca^{2+} increase by PLL was enhanced in the presence of T16A. **B**) Summaries for intracellular basal and PLL-induced peak Ca^{2+}. **C,D**) Whole cell current overlays and corresponding I/V curves in HEK293 cells overexpressing T16A. Experiments were performed in the presence of 0 mM Ca^{2+} and 1 mM BAPTA-AM. Cells were exposed to PLL (100 nM) which did not activate T16A under these conditions. **D)** Summary of current densities in the absence or presence of PLL. Mean ± SEM (number of experiments). #indicates significant difference when compared to mock ($p<0.05$; unpaired t-test).
