# Supplementary material for: KCNE1 does not shift TMEM16A from a Ca2+ dependent to a voltage dependent Cl- channel and is not expressed in renal proximal tubule
Source: Pflugers Arch. 2023 Jul 13;475(8):995–1007. doi: 10.1007/s00424-023-02829-5 (PMC10359377; doi:10.1007/s00424-023-02829-5)
Supplement: Supplementary file 1 — ESM 1 [file 424_2023_2829_MOESM1_ESM.zip › FigS7.pdf]

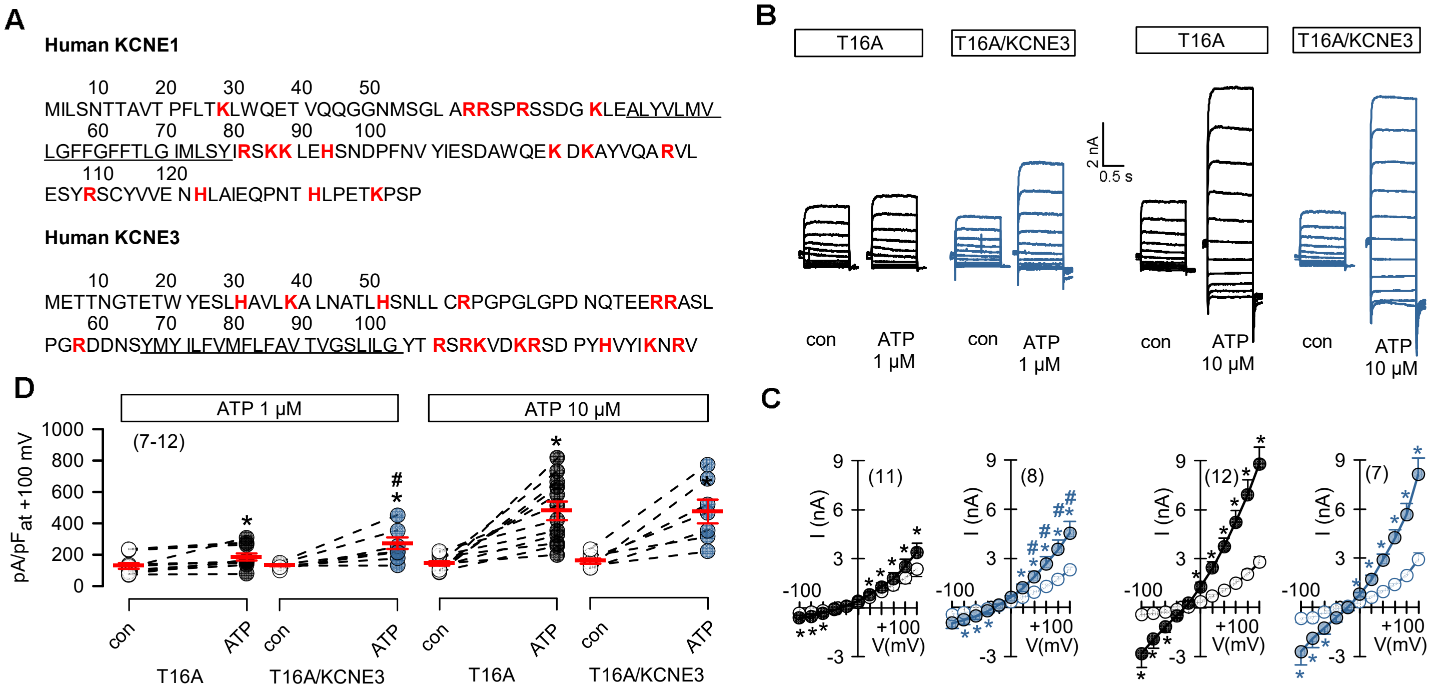

Supplementary Figure 7. *KCNE1 and KCNE3 similarly sensitize TMEM16A towards Ca^{2+}-dependent stimulation.* **A**) Comparison of the amino acid sequence of KCNE1 and KCNE3. **B**) Activation of whole cell currents by ATP (1 µM or 10 µM) in cells expressing T16A only or coexpressing T16A with KCNE3. **C**) Corresponding current/voltage relationships. **D**) Summary of current densities at Vc = +100 mV indicating slightly but significantly enhanced ATP-dependent activation of T16A in the presence of KCNE3. Mean ± SEM (number of experiments). *significant activation by ATP (p<0.05; paired t-test). #significant difference when compared to T16A (p<0.05; unpaired t-test).
