# Supplementary material for: KCNE1 does not shift TMEM16A from a Ca2+ dependent to a voltage dependent Cl- channel and is not expressed in renal proximal tubule
Source: Pflugers Arch. 2023 Jul 13;475(8):995–1007. doi: 10.1007/s00424-023-02829-5 (PMC10359377; doi:10.1007/s00424-023-02829-5)
Supplement: Supplementary file 1 — ESM 1 [file 424_2023_2829_MOESM1_ESM.zip › FigS8.pdf]

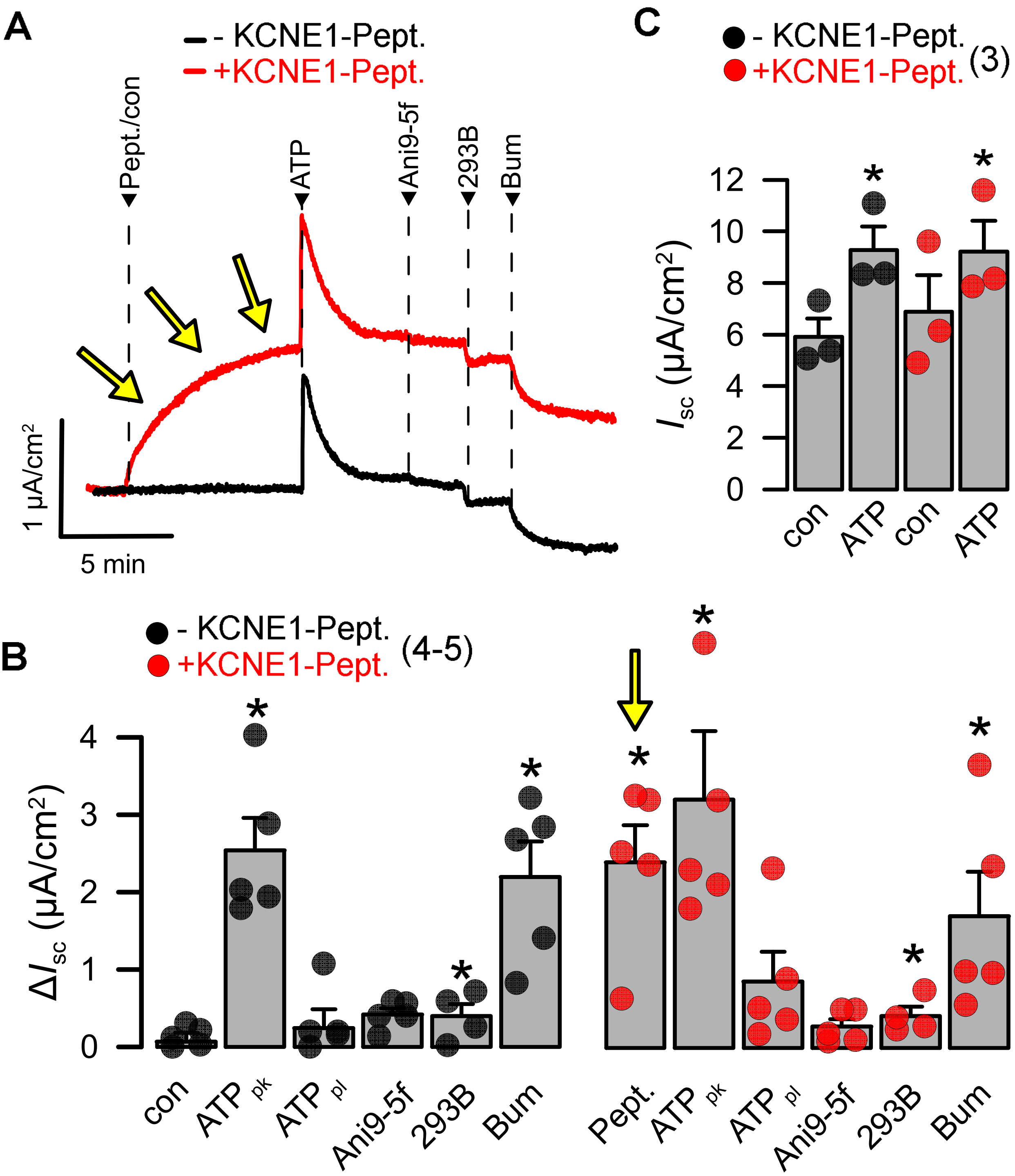

Supplementary Figure 8. *N-terminal KCNE1 peptide causes an offset current in Ussing chamber measurements.* **A**) Original short circuit currents recordings in polarized grown BCi-NS1.1 cells. Application of KCNE1-Pept. (in red, 100 µM, luminal side) causes an offset potential that leads to an offset current (yellow arrows), which is not inhibited by T16A-bocker Ani9-5f (10 µM) or bumetanide (Bum; 50 µM). **B**) Summary of Isc changes induced by KCNE1-Pept., ATP, Ani9-5f, KCNQ1-blocker 293B (10 µM), and Bum. **C**) ATP-activated Isc in the absence or presence of KCNE1-Pept.. Mean ± SEM (number of filters). *indicates significant activation or inhibition ($p<0.05$; paired t-test).
