# Supplementary figures and images for: KCNE1 does not shift TMEM16A from a Ca2+ dependent to a voltage dependent Cl- channel and is not expressed in renal proximal tubule
Source: Pflugers Arch. 2023 Jul 13;475(8):995–1007. doi: 10.1007/s00424-023-02829-5 (PMC10359377; doi:10.1007/s00424-023-02829-5)

Fig. 1E

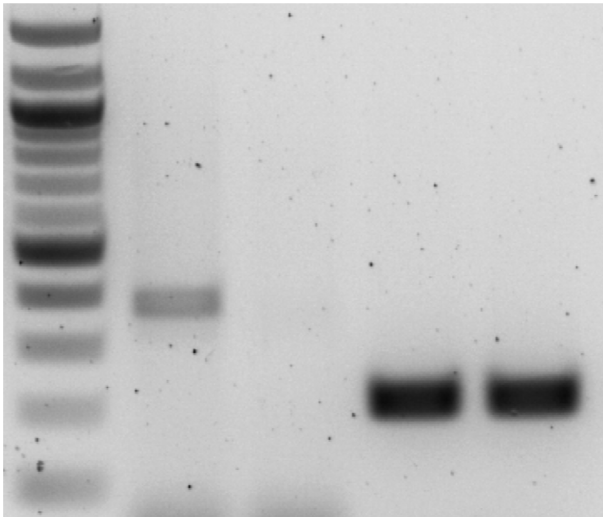

Fig. 3A

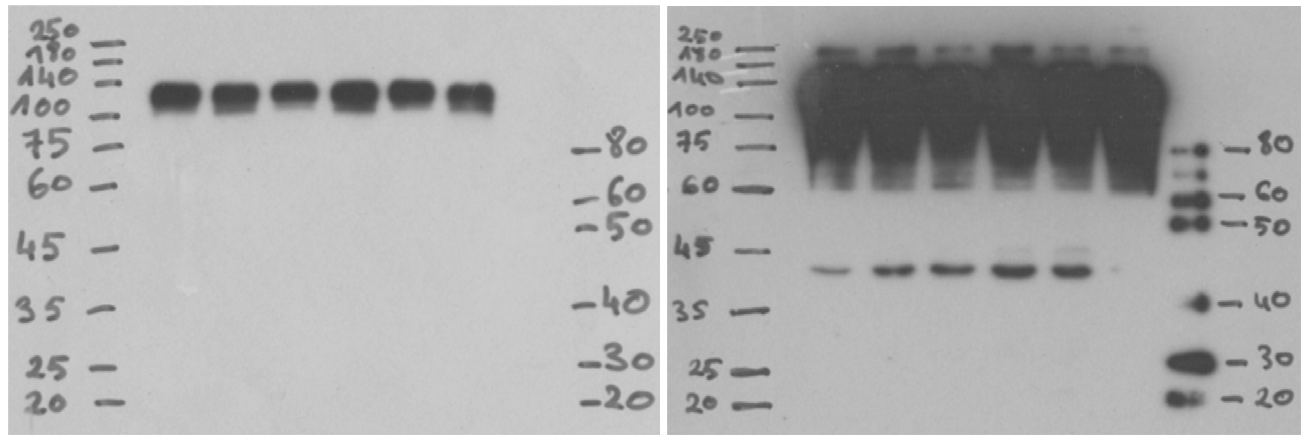

Fig. 6A

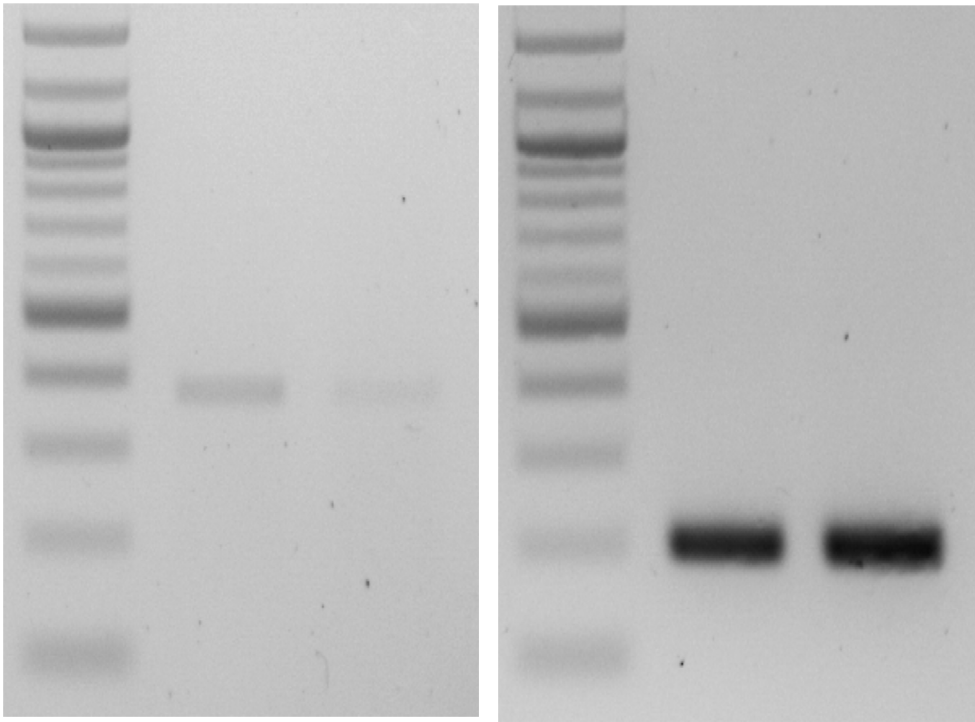

**Supplementary Figure 9: *Uncropped blots.***

Supplement: Supplementary file 1 — ESM 1 [file 424_2023_2829_MOESM1_ESM.zip › FigS9.pdf]
